# Occurrences, sources and health hazard estimation of potentially toxic elements in the groundwater of Garhwal Himalaya, India

**DOI:** 10.1038/s41598-023-40266-7

**Published:** 2023-08-11

**Authors:** R. S. Aswal, Mukesh Prasad, Narendra K. Patel, A. L. Srivastav, Johnbosco C. Egbueri, G. Anil Kumar, R. C. Ramola

**Affiliations:** 1 Department of Environmental Sciences, H.N.B. Garhwal University, Badshahi Thaul Campus, Tehri Garhwal, 249199 India; 2 Chitkara University School of Engineering and Technology, Chitkara University, Solan, Himachal Pradesh India; 3 Department of Earth Sciences, Indian Institute of Technology Roorkee, Roorkee, 247667 India; 4 Department of Geology, Chukwuemeka Odumegwu Ojukwu University, Uli, Nigeria; 5 Department of Physics, Indian Institute of Technology Roorkee, Roorkee, 247667 India; 6 Department of Physics, H.N.B. Garhwal University, Badshahi Thaul Campus, Tehri Garhwal, 249199 India

**Keywords:** Environmental sciences, Natural hazards, Risk factors

## Abstract

High concentrations of potentially toxic elements (PTEs) in potable water can cause severe human health disorders. Present study examined the fitness of groundwater for drinking purpose based on the occurrence of nine PTEs in a heavy pilgrim and tourist influx region of the Garhwal Himalaya, India. The concentrations of analyzed PTEs in groundwater were observed in the order of Zn > Mn > As > Al > Cu > Cr > Se > Pb > Cd. Apart from Mn and As, other PTEs were within the corresponding guideline values. Spatial maps were produced to visualize the distribution of the PTEs in the area. Estimated water pollution indices and non-carcinogenic risk indicated that the investigated groundwater is safe for drinking purpose, as the hazard index was < 1 for all the water samples. Assessment of the cancer risk of Cr, As, Cd, and Pb also indicated low health risks associated with groundwater use, as the values were within the acceptable range of ≤ 1 × 10^−6^ to 1 × 10^−4^. Multivariate statistical analyses were used to describe the various possible geogenic and anthropogenic sources of the PTEs in the groundwater resources although the contamination levels of the PTEs were found to pose no serious health risk. However, the present study recommends to stop the discharge of untreated wastewater and also to establish cost-effective as well as efficient water treatment facility nearby the study area. Present work’s findings are vital as they may protect the health of the massive population from contaminated water consumption. Moreover, it can help the researchers, governing authorities and water supplying agencies to take prompt and appropriate decisions for water security.

## Introduction

Despite being a significant resource, water is needed for everyone to survive on the planet. It is also required for all living beings, like plants. The sustainable development of a country primarily depends on the availability of freshwater sources. Factors such as limited availability, accessibility, and increased contamination of surface water sources are responsible for the dependency of the public on groundwater sources. Moreover, such water supplies are inadequate, particularly during lean seasons, forcing residents to use groundwater for domestic purposes^1^. Like surface water sources, groundwater also covers a significant part of the overall supply in many countries for drinking, domestic, commercial and agricultural purposes. Globally, 70% of withdrawn groundwater is used in the agriculture sector of arid and semi-arid counties^2^. Approximately one-third of human population across the globe mainly relies upon groundwater sources to meet their basic needs of drinking, domestic, agricultural, and commercial uses, etc^3,4^. As a matter of fact, more than 1.5 billion people in the world mainly depend on groundwater sources for drinking^5,6^.

Human activities have affected the quality of various groundwater sources due to the addition of domestic, municipal, agricultural, industrial, outdoor, and commercial pollutants to the environment. Further, groundwater is polluted by various contaminants, out of which the PTEs are the major contaminants^7^. Landfill leachate, application of pesticides, and phosphate fertilizers are some of the anthropogenic activities that can also significantly actuate fluoride contamination in groundwater^8^. In a recent report, out of about 200 million people, more than 50% are affected only in India, China, and Pakistan by mild to severe dental or skeletal fluorosis^4^. As per a previous report, ~ 50–60% of the world's population is suffering from health disorders because of either consumption of polluted water or deficiency in the supply of clean drinking water^9^. From both biological and chemical perspectives, contaminated drinking water may cause severe issues regarding the survival of humans and animals. Unfortunately, groundwater sources are getting adversely affected and enormously polluted occasionally. Hence, many places worldwide are experiencing the adverse effects of groundwater depletion and contamination, including the Indian sub-continent. According to a report, over a billion people are affected due to the unavailability of safe water^10^. Approximately 25,000 people die per annum due to the unavailability of safe water in developing countries^11^. The appraisal of water quality focusing on the prevalence of PTEs and associated health risks has been recently carried out in different parts of the world^12–15^. Studies have also been carried out in the alluvial plains of Northern India, considering the occurrence of anions and cations and associated health implications^16–18^.

In Garhwal Himalaya, Gangotri is the origin place of the Bhagirathi River. In contrast, Yamunotri is famous for originating from the Yamuna River (also known as the sister river of Bhagirathi), later known as Ganga after its confluence with the Alaknanda River at Devprayag). These shrines attract millions of pilgrims and floating tourists every year due to the mythological beliefs of the Hindu religion and the scenic beauty of the locations, respectively. Nevertheless, the Gangotri shrine always has a massive influx of pilgrims and tourists. In addition to the local population, this influx of pilgrims and tourists always has a water requirement for different purposes. The massive influx of pilgrims increases the water requirement in this region. It may pose excellent stress and adverse severe impacts on available limited potable water sources due to the generation of high amounts of municipal trash. Most of the previous studies in the Uttarakhand State have focused on surface water quality evaluation^19,20^ and morphological characteristics^21^ of the hilly terrain of Garhwal and Kumaun Himalaya (India). In addition, some of the studies also considered PTEs occurrence in snow-fed perennial rivers of the Himalayan region^22–25^. Some studies have also evaluated the water quality and health implications of spring sources in various parts of Uttarakhand State^26–30^.

Similarly, the water sources in the plain areas of Uttarakhand State have also been studied with respect to water contamination assessment^31,32^, geochemical characterization, and environmental risk analysis^33^. However, assessing the drinking water quality of groundwater sources of the mountainous Garhwal Himalaya region has not received much attention in the literature. There are currently gaps in knowledge of the groundwater quality for human use in the residential, agricultural and tourist areas of this region. Undoubtedly, a detailed investigation of the occurrences of PTEs in the groundwater sources of this important part of the Himalayan region of India is missing.

Moreover, this region is one of the holiest Hindu places in India, and because of this, the floating population of Hindus is remarkably high. These people depend on such water resources for domestic uses, including drinking water. Hence, to protect public mass, this study may provide a better overview to conduct future relevant research to maintain water quality for domestic purposes. With the above aims, the paper focuses on a comprehensive investigation of the groundwater quality of the Bhagirathi valley region in the Garhwal Himalayan region. In this study, the following objectives are targeted to: (i) analyze the concentrations of PTEs in potable groundwater sources; (ii) spatially analyze the distribution of PTEs in the groundwater; (iii) evaluate pollution levels and water quality for drinking and domestic usages through indexing approaches; (iv) assess non-carcinogenic and carcinogenic health impacts due to the PTEs’ occurrences; and (v) identify the possible sources of contaminants in the groundwater using multivariate statistical analysis. It is hoped that the baseline information on the groundwater quality of this area given in this study would enhance managerial strategies toward effective and sustainable protection of groundwater systems. Also, some cost-effective technologies suggested in this study would ensure the safe removal of PTEs from contaminated water before drinking.

### Sources of PTEs in water

The surrounding environment of available water sources, the efficiency of treatment technologies, treated water quality, constituting materials of water-carrying pipes due to water stagnation, leakage in pipelines, and plumbing pipes (PP) may contribute to the PTEs content in drinking water^34^. The PTEs can also release from soil layers through bio-geochemical processes to the surface water bodies with the help of soil runoff and groundwater sources through leaching and percolation processes^35^. Besides, inorganic fertilizers, surface run-off, partially treated/untreated municipal wastewater, slope factor, land use pattern, excessive withdrawal of groundwater also contaminate drinking water. Further, industrial discharges, applying chemical pesticides, municipal wastewater, and effluents of tanneries are also among the primary sources of Cd, Cu, Ni, Pb, and Cr in potable water^34^. Several PTEs with their source, associated health implications, and guideline values are reported in potable water (Table 1).

**Table 1:** Existence of typical PTEs in water, their sources, and associated health disorders.

PTEs	Source of origin	Affected body organs and system	Guideline value^36^	Reference
Al	Application as a coagulant and mineral weathering of feldspars	Nausea, vomiting, mouth ulcers, skin rashes, skin ulcers, and diarrhea	200*	^37^
Cr	Effluent, tanning, electroplating, and pigment production	Suspected carcinogen, lung tumor, and allergic dermatitis	50	^38^
Mn	Mn-containing agrochemicals, municipal waste water, and sewage sludge	weakness, muscle pain, and slow speech	400	^39^
Cu	Corrosion of distribution pipes and erosion from natural deposits	nausea, vomiting, stomach cramps or diarrhea	2000	^40^
Zn	rock weathering, industrial and domestic wastewater	cardiovascular diseases, hypertension, nausea, and stomach damage	4000*	^40^
As	Industrial wastes, metallic wastes, etc	Endocrine system, hepatic system, and reproductive system	10	^41^
Se	Compounds have dominance of silver, sulphur, copper, lead and nickel	muscle tenderness, tremor, light-headedness, facial flushing, and blood clotting problems	10	^40^
Cd	phosphate fertilizers, and waste incineration	gastric cancer, breast cancer, lung cancer, and renal cancer	3	^42^
Pb	Lead-based batteries, solder, alloys, rust inhibitors, and plastic stabilizers	Anemia, insomnia, headache, dizziness, irritability, weakness of muscles, and renal damages	10	^43^

*Legal limit of water consumption for infants.

### Human exposure to PTEs and associated health risks

PTEs such as Zn, B, Mo, Cu, Fe, and Co at their lower concentrations act as cofactors in various metabolic and other biological processes^35^. The human body requires more than 65 heavy metals, whereas a few others, such as Pb, Hg, Al, As, Cd, Ni are toxic to the human body^44^. PTEs are found at a trace level in the environment. The body cannot metabolize these trace elements and thus can be very toxic in nature^27^. These elements are also characterized as potentially toxic (PTEs) due to associated health risks. Arsenic and cadmium have been recognized as carcinogenic chemical agents for human beings^45^. Moreover, skin and kidney damage are associated with As and Cd exposure, respectively. The adverse human health effects include heart diseases and high blood cholesterol levels due to Sb, anemia due to Pb, renal and liver disorders due to Hg, and Cu is responsible for gastric problems reported by ATSDR^46^ and USEPA^47^. The As, Cd, Pb, Cr, Cu, Hg, and Ni are among various PTEs which can create several human health disorders after their high concentrations through potable water^46^ as arsenic, cadmium, and lead have been extensively studied on risks associated with them on human population^48^.

### Antagonistic and synergistic human health effects

Various international agencies have suggested the threshold concentration values (guideline values) for a few PTEs in potable water to protect the health of human beings^36,47^. The guideline values are estimated based on the lowest concentrations with a non-observed-adverse effect limit (NOAEL). However, the guideline values prescribed by various drinking water regulatory agencies are considered health protective. Nevertheless, the co-exposures of multiple PTEs may also need further attention from the researchers in this issue. The co-exposure to various metals in potable water may change the level of toxicity in users. Due to the prevalence of a toxic element, another metal could show the effect at up to one-twentieth of the prescribed WHO value^49^. Antagonism is a process when the combined effect of two or more elements is lower than the suggested toxic effects of one element. While in the case of synergism, the impact of the combination is more significant than suggested by one element’s toxic effects^50,51^. Therefore, it is important to examine the synergistic or antagonistic consequences of co-exposure to more PTEs via drinking water. In the case of the presence of multiple PTEs in potable water, the impact on human health might be observed below the regulatory limits of respective metals^34^. Despite a low level of a particular element, the combined risk of multiple metals may exceed the allowable risk level. For instance, the risk of As in reference to genotoxicity and metabolism may be intensified due to the co-presence of Sb^52^. In contrast, it has also been observed that the co-exposure to As and Se led to significantly low toxicity^53^. Similarly, Zn is also observed to decrease As toxicity^54^.

## Materials and methods

### Description of the study area

**Figure 1:**
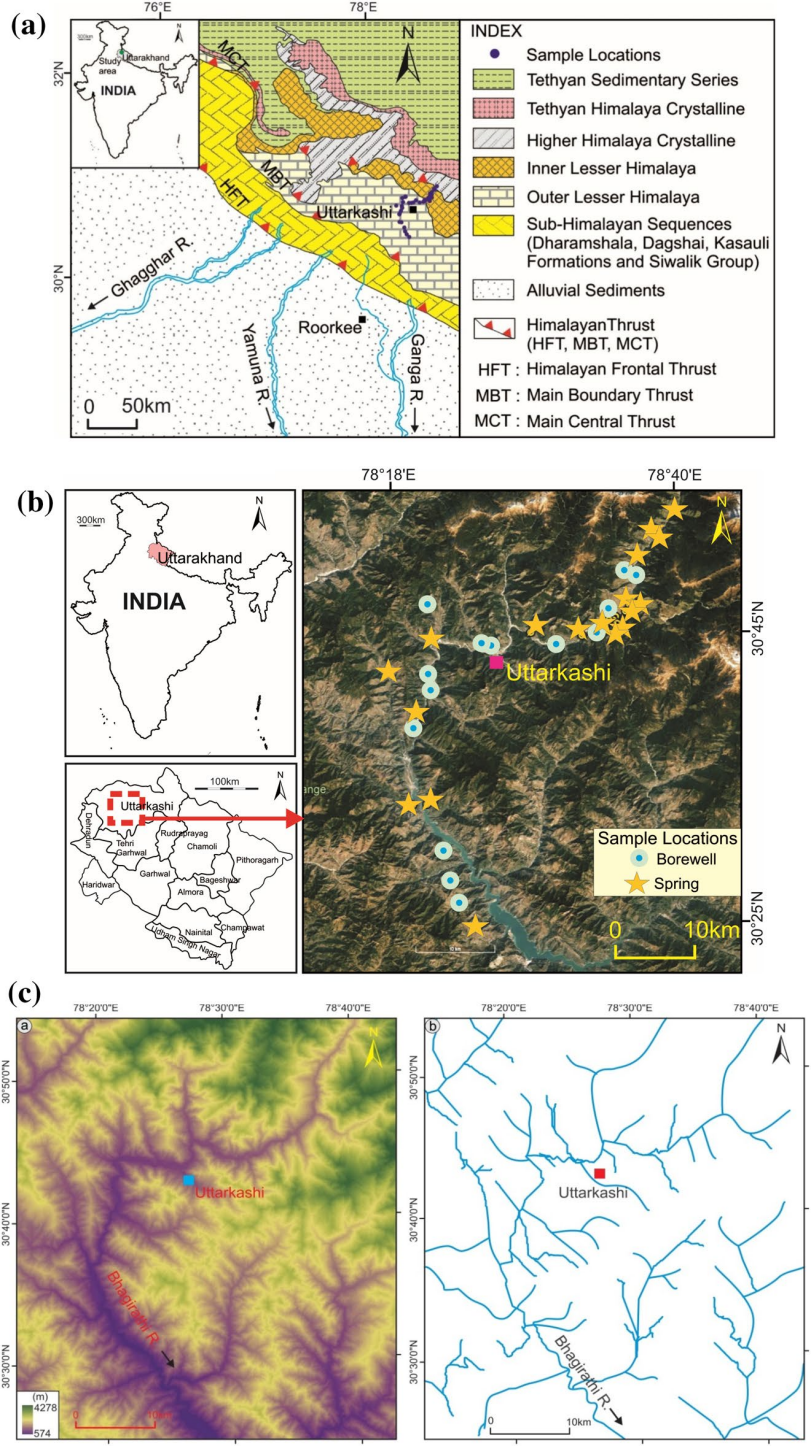
(**a**) Geological map (prepared with ArcGIS, version 10.8.0.12790, URL: https://www.esri.com/en-us/arcgis/products/arcgis-desktop/resources) of the study area. (**b**) Geographical map (prepared with ArcGIS, version 10.8.0.12790, URL: https://www.esri.com/en-us/arcgis/products/arcgis-desktop/resources) of the study area showing sampling sites. (**c**): Map showing the topography and drainage pattern of the study (The map was prepared with ArcGIS, version 10.8.0.12790, URL: https://www.esri.com/en-us/arcgis/products/arcgis-desktop/resources).

The area under the present study lies along the Bhagirathi valley of Garhwal Himalaya, India, approximately 3065 m above the mean sea level. This area is inhabited by the local population living in different patches. Usually, millions of tourists and pilgrims visit the famous Gangotri Dham (located near the study area) every year. These pilgrims generally stay in hotels lying in the Bhagirathi valley region. The ‘Gangotri Dham’ is a famous religious place for Hindu pilgrims near the origin of the Bhagirathi River (Gomukh). Figure 1a and b show the geological pattern and geographical map showing the sampling sites of the study area, respectively. A length of 2500 km of the Himalayan Arc, with a width of 300 km, defines the Tibetan plateau's southern boundary and the Indian-Eurasian collision zone ^55,56^. The collision of the Eurasian and Indian tectonic plates, which began around 55 million years ago, results in the rising of the Tibetan plateau and the formation of the Himalayan orogeny^55,57^. The Garhwal Himalaya is a Himalayan orogeny limited in the north by the Indus-Tsangpo suture and in the south by the HFT, composed of many litho-tectonic units. The dendritic and sub-dendritic drainage patterns are predominant in the studied area. A central trunk stream and lesser tributaries that join it are characteristic of a dendritic drainage pattern in streams, as shown in Fig. 1(c). These tributaries are scattered across the terrain in a manner that resembles the branching structure of tree roots. For instance, local topography and subsurface geology affect the pattern’s shape and texture. The Bhagirathi River is the primary river in the study region.

### Sampling procedure and measurements of trace elements

Thirty-three geographical locations were selected for water sampling in the Bhagirathi valley of Garhwal Himalaya, India, considering the general public's utilization of a particular water source for drinking and other purposes. The samples were collected from bore wells (average depth 30–200 m) and springs. The sampling sites belong to residential and agricultural areas. The samples were taken in PET bottles of one-liter capacity. The sampling bottles were cleaned with 10% (v v-1) HNO_3_ before the sampling, followed by proper rinsing with deionized water. The water samples collected in the pre-processed bottles were further acidified in situ with HNO_3_ (pH < 2). The water samples were brought to the laboratory by maintaining a cold chain (4 °C) for elemental analysis. Inductively coupled plasma-mass spectroscopy (ICP–MS, Make: Perkin Elmer, Model: ELAN DRCe) instrument was used for the determination of the concentrations of different types of PTEs (Al, Cr, Mn, Cu, Zn, As, Se, Cd, and Pb) in water samples.

### Quality control (QC) and quality assurance (QA)

Quality control and quality assurance are critical parameters in the authenticity of experimental data. Therefore, the proper sampling and measurement protocols were followed in different stages of the study. Detailed information about the preparation of water samples for qualitative and quantitative analysis of trace elements, along with calibration verification standard procedures and accuracy of the elemental analysis, are described elsewhere^37^. The detection limits of Al, Cr, Mn, Cu, Zn, As, Se, Cd and Pb are 100, 20, 10, 10, 30, 5, 0.5, 0.1 and 5 mg L^-1^, respectively.

### Indexical analysis for water quality assessment

The fitness of a water source for drinking is defined in terms of permissible limits fora particular PTE by national and international drinking water regulating agencies and concerned governments. Nevertheless, the effects of several non-hazardous and hazardous PTEs in a collective way on human health are not yet defined by the regulatory bodies or governments. Therefore, the potability of groundwater concerning various trace elements is generally assessed by the researchers in terms of pollution indices as given below^58^.

#### Heavy metal pollution index (HPI)

The HPI is defined as the ratio of the sum of the products of the unit weightage factors of different elements and their sub-indices to the sum of unit weightage factors. This index describes the total impact of PTEs on groundwater quality in terms of chemical pollution. The Eqs. (1) and (2) are used to determine the HPI:

1 $$\mathrm{HPI }= \frac{{\sum }_{\mathrm{i}=1}^{\mathrm{n}}{W}_{i}{Q}_{i}}{{\sum }_{\mathrm{i}=1}^{\mathrm{n}}{W}_{i}}$$

2 $$\mathrm{Qi }=\sum_{\mathrm{i}=1}^{\mathrm{n}}\frac{|{M}_{i}-{I}_{i}|}{{S}_{i}-{I}_{i}} \times 100$$

In the present study, the BIS guideline values^59^ for drinking water for PTEs were used in calculations of HPI.

#### Heavy metal evaluation index (HEI)

The HEI describes the overall groundwater quality concerning PTEs concentration and is estimated using the Eq. (3)^60^:

3 $$\mathrm{HEI }= {\sum }_{i=1}^{n}\frac{{C}_{i}}{MAC}$$

#### Contamination index (CI)

The contamination index (CI) is applied to measure the extent of contamination by considering the overall effects of various PTEs assumed to be harmful to an individual^58^. It is computed using Eqs. (4) and (5)^61^. The terminology of different terms used in the above equations is described in Table 2**.**

**Table 2:** Input parameters used in the calculation of HPI, HEI and CI.

S.N	Variable	Nomenclature
1	*N*	Total number of analyzed HMs
2	*W*_*i*_	Unit weightage factor of *i*th HM
3	*S*_*i*_	The maximum permissible limit of *i*th HM
4	*M*_*i*_& C_*i*_	Measured concentration
5	*Q*_*i*_	Sub-index of *i*th HM
6	*I*_*i*_	Maximum desirable limit of *i*th HM
7	*MAC*	Maximum allowed concentration of *i*th HM
8	*CF*_*i*_	Contamination factor of ith HM

4 $$\mathrm{CI}={\sum }_{\mathrm{i}=1}^{\mathrm{n}}\mathrm{CFi}$$

5 $$\mathrm{CFi }= \frac{\mathrm{Ci}}{\mathrm{Si}} -1$$

### Assessment of human health hazards

The risk assessment is commonly characterized as a strategy for determining the potential of any given amount of heavy metals harming human health over a particular period^62^. The risk on human health (may be categorized as non-carcinogenic and carcinogenic) evaluation is often based on an estimate of its related risk level^63^.

#### Non-carcinogenic risk

Non-carcinogenic risk due to the ingestion of analyzed PTEs in potable groundwater was calculated using Eqs. (6) and (7) in terms of Chronic Daily Intake (CDI) through ingestion (CDI_ing_) and dermal (CDI_der_) pathways. Hazard Quotients (HQs) were further estimated for each PTE via ingestion (HQ_ing_) and dermal (HQ_der_) modes using Eqs. (8) and (9), respectively^64,65^ Two population sub-groups, i.e., adults and children considered for risk estimation in this study.

6 $${\mathrm{CDI}}_{\mathrm{ing}-\mathrm{nc}}=\frac{\mathrm{EC}\times \mathrm{DWI}\times \mathrm{EF}\times \mathrm{EP}}{\mathrm{LE}\times \mathrm{BW}}$$

7 $${\mathrm{CDI}}_{\mathrm{der}-\mathrm{nc}}=\frac{\mathrm{EC }\times \mathrm{SA }\times {K}_{p}\times \mathrm{ET}\times \mathrm{EF}\times \mathrm{EP}\times \mathrm{CF}}{\mathrm{LE}\times \mathrm{BW}}$$

8 $${\mathrm{HQ}}_{\mathrm{ing}}=\frac{{\mathrm{CDI}}_{\mathrm{ing}-\mathrm{ncr}}}{{{\mathrm{R}}_{\mathrm{f}}\mathrm{D}}_{\mathrm{ing}}}$$

9 $${\mathrm{HQ}}_{\mathrm{der}}=\frac{{\mathrm{CDI}}_{\mathrm{der}-\mathrm{ncr}}}{{{\mathrm{R}}_{\mathrm{f}}\mathrm{D}}_{\mathrm{der}}}$$

10 $${{\mathrm{R}}_{\mathrm{f}}\mathrm{D}}_{\mathrm{der}}={{\mathrm{R}}_{\mathrm{f}}\mathrm{D}}_{\mathrm{ing}}\times \mathrm{GIAB}$$

Further, the hazard index (HI) for all the analyzed PTEs in potable water can be computed from the following Eqs. (11), (12), and (13):

11 $$HI_{ing} = \sum {HQ_{ing} }$$

12 $$HI_{der} = \sum {\Sigma HQ_{der} }$$

13 $${\text{HI}}_{{{\text{tot}}}} = HI_{ing} + HI_{der}$$

The terminology of different input parameters used in evaluating non-carcinogenic human health hazard risk is given below in Table 3.

**Table 3:** Input parameters used for non-carcinogenic human health risk assessment.

Input parameter	Details of input parameters
EC	Element concentration
DWI	Daily water intake (3.45 L per day^-1^ for adults, for children 2 L day^-1^)
EF	Exposure frequency (365 days year^-1^)
EP	Exposure period (70 years for adults, 10 years for children)
LE	Life expectancy (25,550 days for adults, 3250 days for children)
BW	Body weight (73 kg for adults, 32.7 kg for children)
K_p_	Dermal permeability coefficient (0.001 cm h^−1^)
SA	Exposed skin area (18,000 cm^2^ for adults, 6600 cm^2^ for children)
ET	Exposure time (0.58 h day^-1^ for adults, 1 h day^-1^ for children)
CF	Unit conversion factor (0.001 L cm^-3^)
R_f_D*	Oral reference dose (mg kg^-1^ day^-1^)
	Al (1), Cr (0.003), Mn (0.014), Cu (0.04), Zn (0.3), As (0.003), Se (0.005), Cd (0.0005), and Pb (0.0035)
GIAB**	Gastrointestinal absorption factor
	Al (1), Cr (0.025), Mn (0.04), Cu (1), Zn (1), As (1), Se (1), Cd (0.025), and Pb (1)

*^66^; **^67^.

#### Carcinogenic risk

Continuous ingestion and dermal exposure to certain PTEs in water can potentially bring carcinogenic disorders in humans. The carcinogenic risk (CR) via oral and dermal exposure to PTEs in potable groundwater was evaluated using the Eqs. (14), (15), and (16). It is defined as the incremental risk of a person developing cancer over their lifetime exposure to a possible carcinogen^65,68^. The CR_ing_ and CR_der_ were estimated for As, Cr, Cd, and Pb in water using the following equations:

14 $${CR}_{ing-cr}={CDI}_{ing-ncr}\times {SF}_{ing}$$

15 $${CR}_{der-cr}={CDI}_{der-ncr}\times {SF}_{der}$$

16 $${SF}_{der}=\frac{{SF}_{ing}}{GIABS}$$

The cancer slope factors (CSFs) values of 0.5, 1.5, 0.0061, and 0.0085 were used to compute CR_ing_ and CR_der_ for As, Cr, Cd, and Pb, respectively.

## Results and discussions

### Occurrence of PTEs in the groundwater of the study area

**Figure 2:**
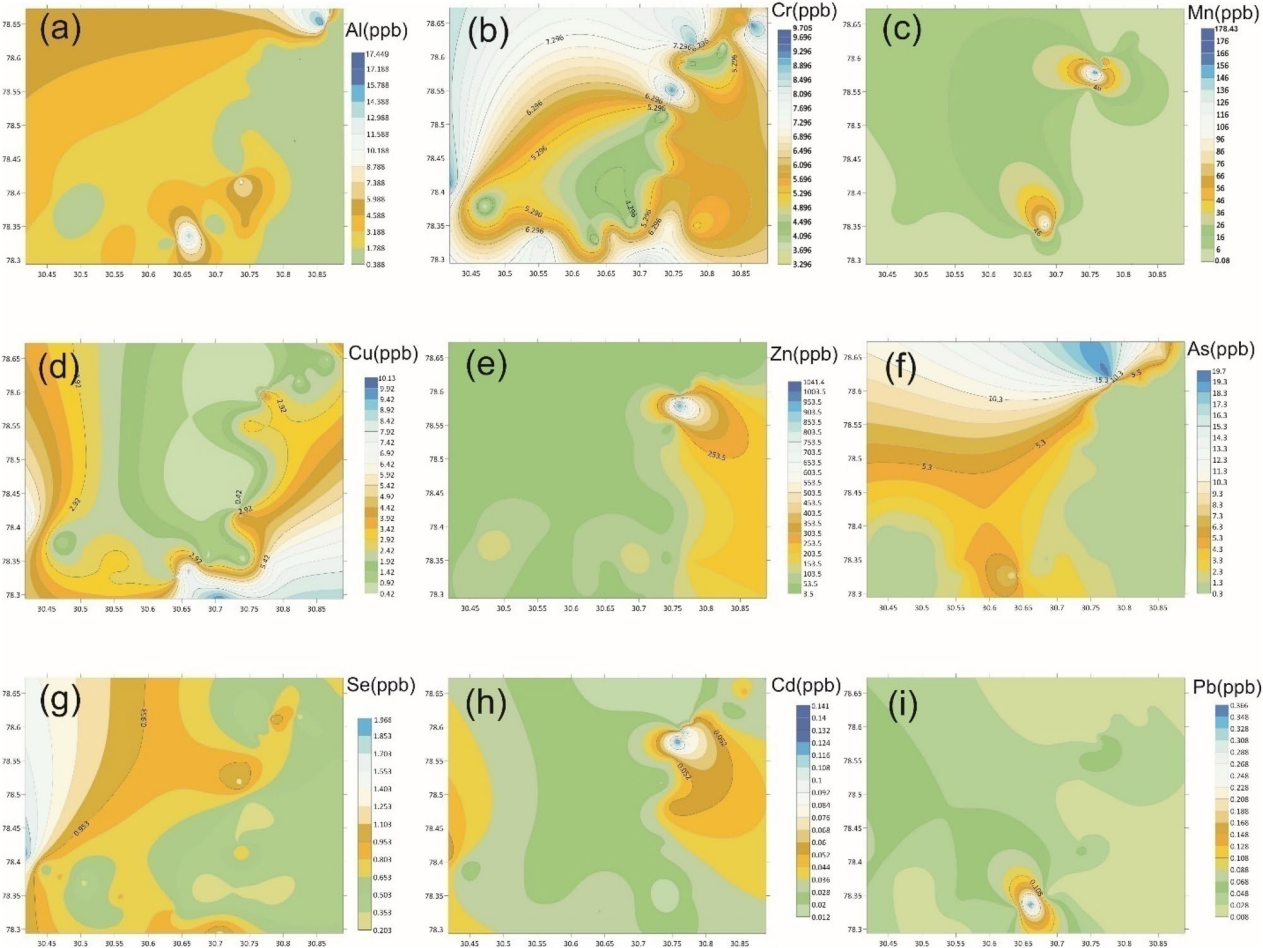
Distribution of PTEs in groundwater of Bhagirathi valley, Garhwal Himalaya.

The statistical information about analyzed PTEs estimated in different groundwater sources in Bhagirathi Valley is depicted in Table 4. The contents (in μg L^−1^) of the analyzed PTEs in different water sources were found in the order: Zn (1041.436) > Mn (178.443) > As (19.734) > Al (17.449) > Cu (10.138) > Cr (9.705) > Se (1.966) > Pb (0.366) > Cd (0.141).The contour maps created in ArcGIS 10.8 showing the variation of analyzed PTEs contents over the sampling sites are demonstrated in Fig. 2a–i. The point dataset of PTEs concentration was imported into ArcGIS software to prepare a contour map, and Spatial Analyst extension was enabled in it. The Kriging interpolation method was used to create a continuous raster surface from the point data because it produces the best illustrations of spatially distributed data.

**Table 4:** Distribution of PTEs in groundwater samples of Bhagirathi valley, Garhwal Himalaya.

Heavy metal	Acceptable limit (µg/L)		Min	Max	A.M	S.D	Ske	Kur	% of water samples above Acceptable Limit	
	BIS^59^	WHO^36^							BIS^59^	WHO^36^
Al	30	200^a^	0.33	17.45	3.09	3.85	2.60	7.16	N.A	N.A
Cr	50	50	3.23	9.71	5.86	1.79	0.84	− 0.03	N.A	N.A
Mn	100	400	0.08	178.44	13.31	38.03	3.50	12.35	~ 9	N.A
Cu	50	2000	0.42	10.14	2.79	2.56	1.30	1.05	N.A	N.A
Zn	5000	4000^a^	3.57	1041.44	135.72	217.40	2.91	9.51	N.A	N.A
As	10	10	0.31	19.73	3.19	3.79	2.92	10.91	~ 4	~ 4
Se	10	10	0.20	1.97	0.64	0.37	1.61	3.87	N.A	N.A
Cd	3	3	0.01	0.141	0.04	0.03	2.37	5.17	N.A	N.A
Pb	10	10	0.01	0.366	0.04	0.06	4.71	24.72	N.A	N.A

Min: Minimum, Max: Maximum, A.M.: Arithmetic Mean, S.D.: Standard Deviation, Var: Variance, Ske: Skewness, and Kur: Kurtosis, and ^a^Legal limit for water intended for infant consumption.

The Al, Cr, Cu, Zn, Se, Cd, and Pb concentrations were not found to exceed the corresponding acceptable limits recommended by regulatory agencies for drinking water. The sources of origins and possible human health effects of these PTEs are portrayed in Table 1.

Al concentration in studied groundwater was not observed to be more than the acceptable limit prescribed by BIS or WHO. All the water sources were observed safe with respect to Cr contamination according to the guidelines of BIS and WHO. Mn concentration exceeded the BIS limit of 100 µg/L in 9% of analyzed water samples.None of the samples was observed to have Mn concentration exceeding WHO guideline value of 400 µg/L (Table 4). Thus, contamination due to Mn is almost negligible in the region. The Cu and Zn values were observed to be relatively lower than those reported in previous investigations in Uttarakhand, as shown in Table 5. The concentrations of Mn and Asin in the analyzed groundwater samples exceeded their respective BIS acceptable limits in 9% and 4% samples, respectively (Table 4). The analyzed groundwater samples of Bhagirathi valley were observed to show Se well below the acceptable limit of 10 µg/L^36,59^. The concentrations of Cd and Pb in the analyzed groundwater samples were also found to lie below their safe limits.

**Table 5:** Comparison of PTEs concentration (µg/ L) in water sources of the present study with other relevant recent studies (last 5 years) carried out in Uttarakhand (India).

S.N	Location/Details	Al	Si	Cr	Mn	Fe	Co	Ni	Cu	Zn	As	Se	Cd	Ba	Hg	Pb	Reference
		µg/L															
1	Kumaun Himalaya/Naini Lake basin (Spring)	NA	NA	NA	10–150	BDL-210	NA	NA	BDL-240	10–150	NA	NA	NA	NA	NA	NA	^27^
2	Haridwar/ Surface water	NA	NA	NA	NA	90–780	NA	NA	NA	NA	NA	NA	NA	NA	NA	NA	^73^
3	Garhwal-Himalaya region/Surface water	NA	NA	NA	NA	4101- 6562	NA	NA	2- 10	12–59	NA	NA	NA	NA	NA	1- 10	^74^
4	Haridwar/Surface water	1.38–2.42	47.40–58.00	NA	2.57- 4.51	5222.56- 5696.92	2.39–6.35	2.39–2.91	2.98- 14.51	32.83- 42.68	NA	NA	2.42–2.83	NA	NA	2.23- 5.48	^75^
5	Haridwar/Surface water	1.217–2.842	2.733–49.417	NA	2.808–6.225	5289.467–5496.525	2.575–4.258	2.233–48.583	3.183–4.358	26.233–39.433	NA	NA	1.642–3.367	NA	NA	2.900–6.917	^76^
6	Rishikesh and Haridwar/Surface water	NA	NA	NA	NA	110- 350	NA	NA	NA	1140- 1140	NA	NA	NA	NA	NA	NA	^77^
7	Haridwar/Groundwater	NA	NA	BDL-5.39	0.19–2086	30–9280	NA	0.04–129	0.03–50.9	NA	0.10–102	NA	BDL-1.31	NA	NA	BDL-36.8	^32^
8	Uttarakhand/Surface water	NA	NA	0.571–7.171	NA	NA	NA	0.734–23.280	0.319–1.257	0.498–9.632	2.511–16.142	NA	0.004–0.177	NA	5.164–7.820	0.008–0.933	^78^
9	Uttarakhand/Groundwater	0.067–27.4	NA	0.259–4.5	0.001–140	NA	0.017–0.91	0.41–5.5	0.072–9.3	5.188–4164	NA	NA	0.001–0.9	NA	NA	0.003–11.3	^26^

BDL- Below detection limit; NA- Not available.

In brief, the concentrations of analyzed PTEs (except Mn and As) in all the analyzed water samples were found below the safe limits suggested by various drinking water regulating agencies. Further, the pollution level of Mn and As in groundwater sources is marginal in the study area. More accurately, the concentrations of other PTEs except for Mn and As in this study are observed far below the acceptable limits, ensuring that groundwater is safe for drinking (Table 4). However, these findings are inconclusive, and comprehensive coverage of more water sources may provide a representative picture of the PTEs occurrence in different kinds of water bodies in the study area.

### Comparison of PTEs concentrations with other studies

The findings of the current work were compared with other studies in different water sources of the mountain and plain areas of the Uttarakhand state (Table 5). Gaur et al. reported elevated concentrations of Al, Cr, Cd, and Pb in the groundwater of Haridwar and Dehradun districts, Uttarakhand, with the range of 56–58, 94–98, 130–133, and 84–90 µg L^−1^, respectively^69^. Similarly, Cu and Pb contents were also reported to be very high in the range of 140–240 and 240–340 µg L^−1^, respectively, in the Mallital portion of Spring-Fed Naini Lake^70^. The concentration of Cu fluctuated between BDL-200 and BDL-125 µg L^−1^ in the Eastern and Western basins of Sattal Lake of the Nainital district, which is under the safe limit ^71^. The concentration of Cd in Naini Lake of the Nainital region was reported by CGWB^72^ up to 1000 μg L^−1^, which is several times above than guideline limit of 3 μg L^−1^.

In the research by Chhimwal et al^27^, the Mn concentration in groundwater of the Naini lake basin was noted in a range from 10 to 150 μg L^−1^, which is above than acceptable level of 100 μg L^−1^. The same study also obtained even high Cu concentrations ranging from BDL (below detection limit) to 240 μg L^−1^. Similarly, Cu, among other analyzed trace metals, was observed above the desirable limit of BIS (2012) in the groundwater of the Haridwar district and ranged from BDL to 73 μg L^−1^^79^. Matta et al.^76^ have also reported the high occurrence of Ni and Cd contents in the Haridwar region ranging from 2.233–48.583 and 1.642–3.367 μg L^−1^ compared to other analyzed PTEs in the surface water system. Haritash et al.^80^ also reported elevated Ni (BDL-36.7 μg L^−1^) and Cu (32.1–58.1 μg L^−1^) concentrations in the surface water of the Rishikesh region. Similarly, measurements for Mn, Ni, Cu, As, and Pb were also observed to be very high except for Cu and fluctuated in the range of 0.19–2086, 0.04–129, 0.03–50.9, 0.10–102, and BDL-36.8 µg L^−1^, respectively by Khan and Rai ^32^ in the groundwater sources of Haridwar district. The exceeded contents of Ni, As, and Hg in the surface water of Garhwal region, Uttarakhand, were reported by Kumar et al.^78^ with a range of0.734–23.280, 2.511–16.142, and 5.164–7.820 μg L^−1^, respectively. Among these elevated concentrations, only Hg was observed to be several times higher than the safe limit of 1 μg L^−1^. The concentrations of Mn and Pb in groundwater sources were previsouslynotedas0.001–140 and 0.003–11.3 μg L^−1^, respectively^26^. Thus, most of the surface and groundwater samples exceeded the safe limit of various analyzed PTEs. It is also observed that compared to other studies, the results of the present study show lower concentrations of PTEs except for Mn and As in drinking water sources compared to their corresponding acceptable and permissible limits advocated by various national and international regulating agencies. It indicates that although the possibility of significant risk due to the above-mentioned PTEs are less, the public drinking water supplying agencies need to act on effective treatment process, exploring alternate water sources, or regular monitoring of the water supply schemes in the Bhagirathi valley of Garhwal Himalaya in India.

**Figure 3:**
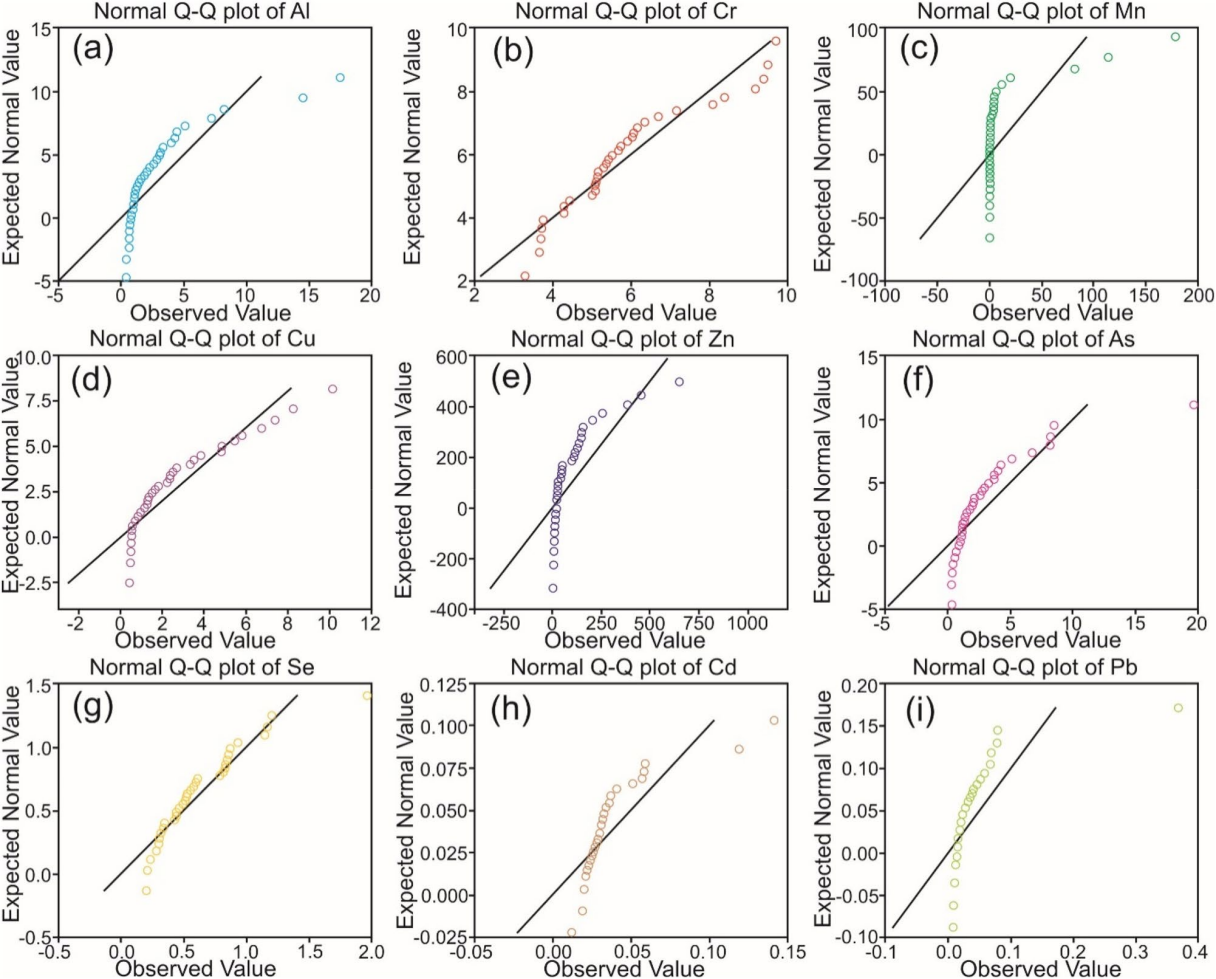
Q-Q Plots for PTEs in water sources of Bhagirathi valley: (**a**) Al, (**b**) Cr, (**c**) Mn, (**d**) Cu, (**e**) Zn, (**f**) As, (**g**) Se, (**h**) Cd, and (**i**) Pb.

The quantile–quantile (Q-Q) plots for the observed water quality data are shown through Fig. 3a–i. It shows non-normal behaviour of all analyzed PTEs concentration in groundwater sources. It indicated that 8 out of 9 analyzed metals (such as Al (Ҝ = 7.156), Mn (Ҝ = 12.348), Cu (K = 1.047), Zn (K = 9.514), As (Ҝ = 10.918), Se (Ҝ = 3.865), Cd (Ҝ = 5.166), and Pb (Ҝ = 24.716) were found as Leptokurtic behaviours with heavy-tails. However, only Cr (Ҝ =  − 0.029) exhibit Platykurtic behaviour with flat tails. All analyzed PTEs concentrations in groundwater of Bhagirathi valley demonstrated positively skewed (Al = 2.609, Cr = 0.841, Mn = 3.500, Cu = 1.303, Zn = 2.909, As = 2.923, Se = 1.603, Cd = 2.366, and Pb = 4.705) frequency distribution signifying their non-normal behaviour.

**Figure 4:**
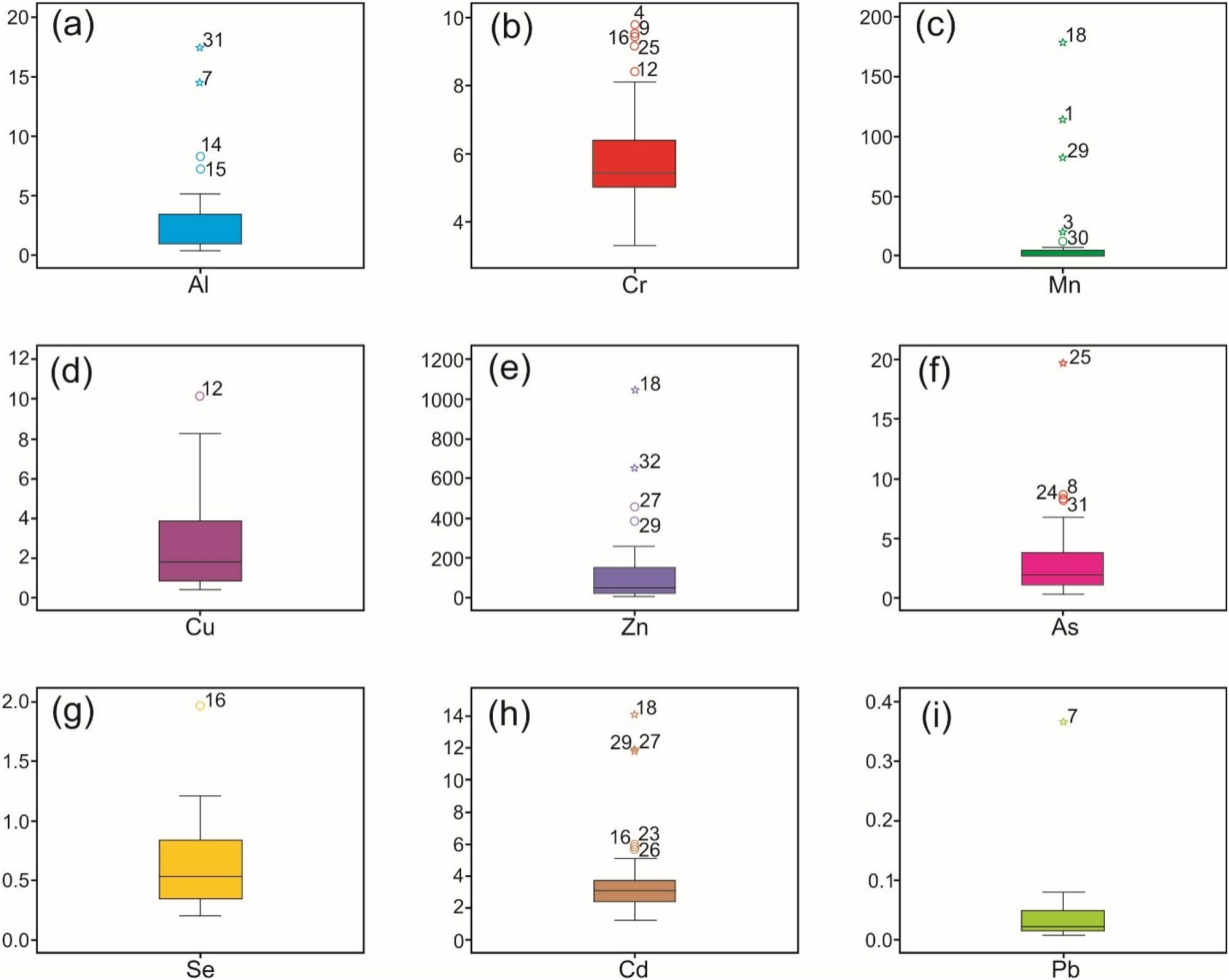
Box and whisker plots of analyzed metals in groundwater sources of Bhagirathi valley: (**a**) Al, (**b**) Cr, (**c**) Mn, (**d**) Cu, (**e**) Zn, (**f**) As, (**g**) Se, (**h**) Cd, and (**i**) Pb.

The Box and Whisker plots for the obtained dataset are shown in Fig. 4a–i. It points out that Mn and Zn have been detected to have higher values than other evaluated PTEs. These plots display umpteen outliers in all analyzed PTEs due to concentration disparity.

### Correlation analysis for contaminant source apportionment

In general, the interpolation methods may not accurately predict the variability of PTEs concentration. Therefore, applying a suitable correlation method is imperative for a better understanding of PTEs variation and source apportionment^4^. Spearman correlation analysis based on the non-normal distribution of the dataset was further calculated for the determination of particular PTE(s) in an analyzed groundwater sample which helps to know the prevalence of another PTE(s) along with their coexistence due to anthropogenic reasons in the studied area^81^. Table 6 demonstrates the correlation test with statistical significance between analyzed PTEs. The dependency was found between PTEs displayed in Table 6, in which Spearman coefficient ‘r’ of Al against Cr is **0.508** at* p *= 0.003, showing dependence between Al and Cr. Similarly, the coefficient ‘r’ of Mn against Zn (**0.741**,* p *= 0.000); and Cu against Pb (**0.599**,* p *= 0.000) exhibits strong (> 0.50) by Bangotra et al^81^ but a positive relationship between (Al—Cr), (Mn –Zn), and (Cu—Pb). However, Spearman coefficient ‘r’ of Zn against Cd (**0.460** at* p *= 0.007); and Se against Pb (**0.478** at* p *= 0.005) indicates low (< 0.50) and positive correlation between the pairs of Zn and Cd, and Se & Pb. In contrast, the negative coefficient ‘r’ of Al against Mn (**− 0.578**,* p *= 0.000) and Zn (**− 0.646**,* p *= 0.000); Cr verses Mn (**− 0.575**,* p *= 0.000), Zn (**− 0.540**,* p *= 0.001) shows a strong correlation between (Al–Mn), (Al–Zn), (Cr–Mn), and (Cr–Zn). Consistent with the line above, Mn established an opposite weak correlation with As (**− 0.441**,* p *= 0.010). Therefore, the above Spearman correlation test revealed that variation of one of the analyzed PTEs showed a corresponding change in another. However, the nonappearance of correlations between any two different PTEs suggests that a single factor is not responsible for the control of concentrations of such PTEs, a combination of different phases supported by various geochemical substances may be responsible^81^.

**Table 6:** Spearman correlation analysis for PTEs in potable groundwater samples of the Bhagirathi valley region.

		Al	Cr	Mn	Cu	Zn	As	Se	Cd	Pb
Al	Correlation Coefficient	1.000	0.508**	− 0.578**	0.292	− 0.646**	0.303	0.192	− 0.107	0.156
	Sig. (2-tailed)		0.003	0.000	0.099	0.000	0.086	0.284	0.554	0.386
Cr	Correlation Coefficient		1.000	− 0.575**	0.244	− 0.540**	0.203	0.250	0.002	0.118
	Sig. (2-tailed)			0.000	0.172	0.001	0.258	0.161	0.990	0.512
Mn	Correlation Coefficient			1.000	− 0.129	0.741**	− 0.441*	− 0.175	0.294	− 0.159
	Sig. (2-tailed)				0.473	0.000	0.010	0.329	0.097	0.376
Cu	Correlation Coefficient				1.000	− 0.164	− 0.163	0.303	0.151	0.599**
	Sig. (2-tailed)					0.360	0.363	0.086	0.400	0.000
Zn	Correlation Coefficient					1.000	− 0.329	− 0.247	0.460**	− 0.231
	Sig. (2-tailed)						0.061	0.165	0.007	0.196
As	Correlation Coefficient						1.000	0.155	− 0.167	− 0.064
	Sig. (2-tailed)							0.389	0.354	0.725
Se	Correlation Coefficient							1.000	0.083	0.478**
	Sig. (2-tailed)								0.646	0.005
Cd	Correlation Coefficient								1.000	0.071
	Sig. (2-tailed)									0.696
Pb	Correlation Coefficient									1.000
	Sig. (2-tailed)									

*n* = 33 (number of samples); **Correlation is significant at the 0.01 level (2-tailed); *Correlation is significant at the 0.05 level (2-tailed); and Bold value indicates strong correlation.

The significant positive and negative correlations between various elements in the groundwater data point towards potential sources of contamination that may have both natural and anthropogenic origins. For instance, the positive correlation between Al and Cr suggests a common source of pollution, such as industrial activities or natural weathering of geological formations rich in both elements. Chromium is often associated with various industrial processes, such as metal plating, leather tanning, and stainless-steel production^82,83^. Aluminum can also stem from industrial activities, but it is also naturally present in the Earth's crust^84^. Thus, the nearby industrial sites and geological formations in the study area are potential sources of contamination. The positive correlation between Zn and Mn indicates a possible relationship between these two elements' sources. Zinc contamination can arise from industrial activities, including metal smelting, galvanization, and battery production^85,86^. Manganese contamination can also result from industrial discharges or runoff from agricultural activities^87^. Both elements might originate from common anthropogenic activities or geological sources, necessitating further investigations into the potential industrial and agricultural activities in the vicinity of the groundwater sites. The significant positive correlations between Cd and Zn, as well as Pb and Cu, could be indicative of similar pollution sources. Cadmium is commonly found in batteries, pigments, and industrial processes such as metal plating^82,88^. It can contaminate the environment alongside zinc, which is also utilized in galvanization and other industrial applications. Similarly, copper and lead often co-occur in industrial settings, including plumbing, electronics, and mining^82,88,89^. These correlations suggest that anthropogenic activities in the area may be contributing to the co-contamination of these elements in the groundwater.

On the other hand, the observed significant negative correlations between certain elements in the groundwater data also provide important clues about potential contamination sources. The negative correlations between Mn and Cr, Mn and Al, Zn and Al, Zn and Cr, and As and Mn suggest contrasting sources or processes that influence their concentrations in the groundwater^83,88^. The negative correlation between Mn and Cr and Mn and Al may indicate that different industrial activities or geological sources are contributing to the presence of these elements in the groundwater. For example, while Cr may arise from industrial processes like metal plating, Mn could originate from the natural weathering of rocks or agricultural runoff^82,87,88^. Aluminum, on the other hand, might come from both industrial sources and natural occurrences. Similarly, the negative correlations between Zn and Al, Zn and Cr, and As and Mn also suggest the possibility of diverse sources affecting the concentrations of these elements. Arsenic contamination is often linked to natural sources like geothermal activity or mineral deposits^89^, whereas Zn and Al contamination can have both natural and anthropogenic origins^83,88^.

### Principal component analysis for contaminant source apportionment

**Figure 5:**
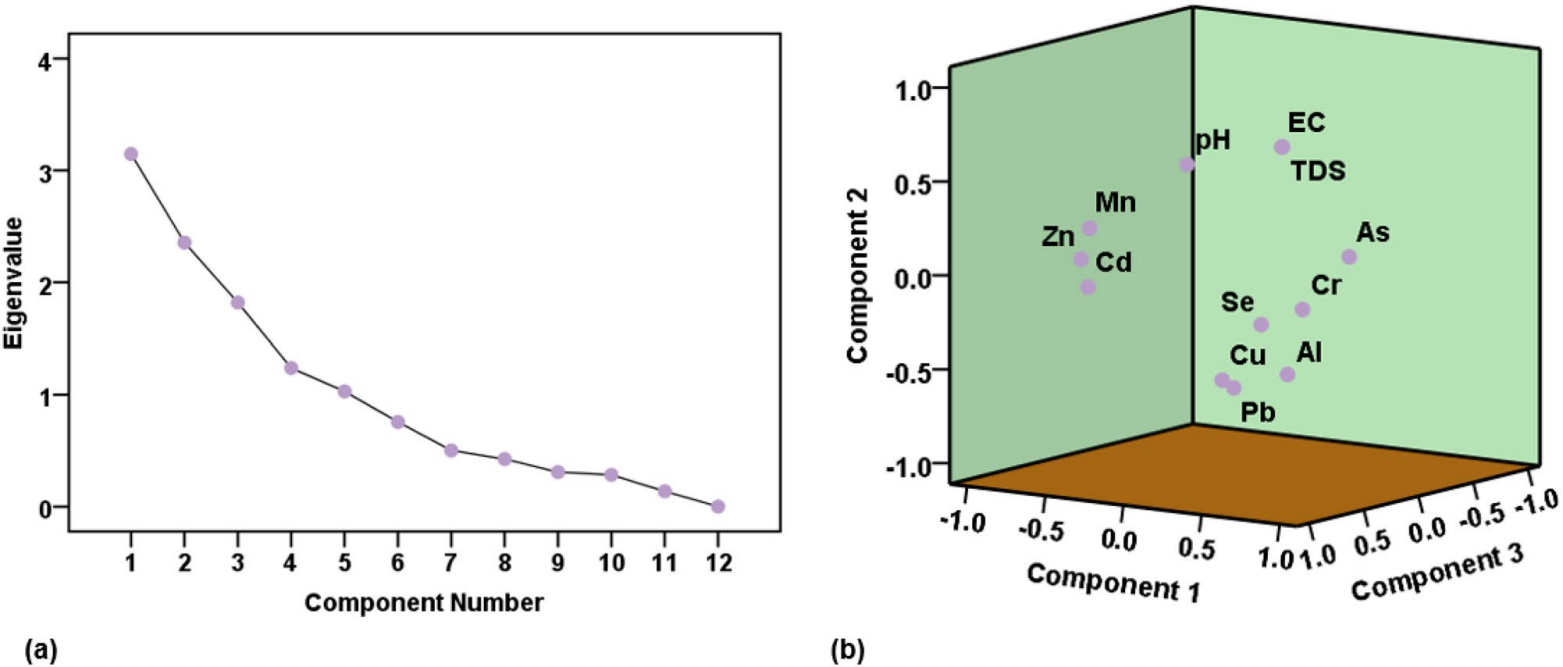
(**a**) Scree plot for component selection and (**b**) 3D spatial distribution pattern of the components.

The principal component analysis (PCA) was performed on the groundwater quality data, in order to complement the results of Spearman's correlation analysis, using IBM SPSS software (version 22). The scree plot of the analysis and the 3D visualization of the principal components are presented in Fig. 5. On the other hand, the principal component extractions, alongside their percentages of variance and eigenvalues, are shown in Table 6. Five principal components with eigenvalues greater than 1 were selected. Cumulatively, they explained about 79.915% of the variabilities in the groundwater quality. The PC 1, explaining 26.226% variance, has significant negative loadings on Mn, Zn, and Cd. However, Cr had significant positive loading in the PC 1. The PC 2, which had 19.226% variability, has significant positive loadings on pH, EC, and TDS. However, Al and Pb were negatively loaded in PC 2 (Table 6). In PC 3, with percentage of variance of 15.160%, Cu has positive loading whereas As has negative loading. In PC 4 (variability = 10.320%), Pb was negatively loaded while Se was positively loaded. Finally, the PC 5, with 8.577% variance, has significant positive loading on As (Table 7).

**Table 7:** Principal component extractions and their loadings.

	PC 1	PC 2	PC 3	PC 4	PC 5
pH	− 0.115	**0.583**	0.365	− 0.340	0.166
EC	0.495	**0.738**	0.371	0.016	0.029
TDS	0.497	**0.740**	0.366	0.016	0.028
Al	0.377	** − 0.515**	0.150	** − **0.443	0.266
Cr	**0.511**	** − **0.149	0.203	0.429	0.494
Mn	** − 0.747**	0.178	0.353	** − **0.022	0.250
Cu	0.301	** − **0.484	**0.636**	0.029	− 0.115
Zn	** − 0.884**	** − **0.017	0.236	0.138	0.123
As	0.312	0.008	**-0.507**	0.132	**0.679**
Se	0.328	** − **0.232	0.320	**0.698**	− 0.231
Cd	** − 0.726**	** − **0.127	0.397	0.232	0.267
Pb	0.278	** − 0.547**	0.498	** − 0.404**	0.093
Eigenvalue	3.147	2.356	1.819	1.238	1.029
% of Variance	26.226	19.632	15.160	10.320	8.577
Cumulative %	26.226	45.859	61.018	71.338	79.915

Significant values are in bold.

In addition to the correlation analysis, the results of the PCA provide valuable insights into the associations between the groundwater quality parameters and their potential contamination sources. In the first principal component (PC 1), the significant negative loadings of Mn, Zn, and Cd suggest that these elements may have a common contamination source. The positive loading of Cr indicates a separate source of contamination for this element in the PC 1. This differentiation in loadings suggests that there might be natural geological processes or anthropogenic activities contributing to the presence of Mn, Zn, and Cd in the groundwater, while Cr might be associated with a distinct pollution source. Moving to the second principal component (PC 2), the positive loadings of pH, EC, and TDS indicate a common association between these parameters, possibly related to natural mineral dissolution or leaching processes^90,91^. Conversely, negative loadings of Al and Pb suggest that they might have a different contamination source. The presence of Al and Pb in groundwater can often be linked to industrial discharges, mining, agricultural runoff, or urban activities^82–84,88^.

Similarly, the associations observed in PC 3, PC 4, and PC 5, point to unique contamination patterns for Cu, As, Pb, Se, and As. Cu's positive loading in PC 3 could be attributed to industrial or agricultural activities^82,87,89^, while As's negative loading may indicate its presence from natural geological sources^89^. Pb's negative loading in PC 4 suggests a potential anthropogenic origin, possibly from industrial practices or old water supply infrastructure^90,91^. Meanwhile, Se’s positive loading in PC 4 could be linked to natural geological processes^89^. Finally, the significant positive loading of As in PC 5 could be associated with a specific natural or anthropogenic contamination source in the area. Geological formations rich in arsenite have been reported to contribute to enrichment of groundwater with arsenic^89^. Human activities like mining also play important role in arsenic contamination^89^. Overall, the findings of the PCA provided crucial information for understanding the contamination sources of various groundwater quality parameters. The separation of loadings across different principal components suggested that multiple sources, both natural (geological) and anthropogenic, contributed to the overall groundwater quality variabilities. Therefore, more investigations may be necessary to pinpoint the exact sources of these PTEs in the groundwater and subsequently implement effective measures for contamination prevention and remediation.

### Human health risk assessment

#### Pollution Indices

The estimated pollution indices represent the combined effects of PTEs on quality of studied water sources and such methods are globally recognized used in the comprehensive water quality evaluation of different potable water sources^58,61^. The HPI, HEI, and CI were determined separately during this study for each sample site by incorporating individually measured concentrations of analyzed PTEs and using their corresponding standard limits (Table 4). The value of HPI was found to range from 0.117 to 4.233 with a mean of 1.354 ± 0.922. The indices (Table 8) showed that HPI for all the drinking water sources were far below the critical limit of 100 reported by Prasad and Bose^92^.

**Table 8:** Classification of analyzed potable water based on calculated HPI, HEI and CI values (n = 33).

Pollution indices	Classification	Suitability of water	Category-wise contribution of samples	Percentage contribution	Critical value	Reference
HPI	< 25	Excellent	33	100	**100**	^93^
	26–50	Good	Nil	N.A		
	51–75	Poor	Nil	N.A		
	76–100	Very poor	Nil	N.A		
	> 100	Unsuitable	Nil	N.A		
HEI	< 10	Low	33	100	**20**	^94,95^
	10–20	Medium	Nil	N.A		
	> 20	High	Nil	N.A		
CI	< 1	Low	33	100	**3**	^94^
	1–3	Moderate	Nil	N.A		
	> 3	High	Nil	N.A		

*N.A.- Not Applicable.*

Threshold values are in bold.

There is an inverse relationship between computed HEI and water quality. It means that lower HEI affirms the better quality of the water source. The mean value of HEI was computed as 0.333 ± 0.144 with a marginal range between 0.150 and 0.870 for the analyzed water sources. The obtained present level of HEI shows that water quality falls far within the first category of the low pollution range. Moreover, the CI was used as a reference to estimate the extent of overall metal pollution in the concerned water source^58^. The estimated CI may further be grouped into three categories as follows: CI < 1 (low), Cd = 1–3 (moderate) and Cd > 3 (high). The mean CI value was  − 8.667 ± 0.144 with marginal fluctuation from -8.850 to -8.130 for water sources. The indices showed that CI for all the potable water sources were observed below the critical limit of 3 reported by Backman et al^58^.This suggests that high-quality water for drinking about the analyzed PTEs in the studied region of Bhagirathi valley region. The computed carcinogenic risk assessments have good stability and indicate a unique and natural source of PTEs in the study area, somewhat related to the soil-geochemical background than to the anthropogenic origin of these analyzed PTEs. Present study demonstrated a ‘nil to very low’ degree of water contamination concerning the analyzed PTEs. Thus, the studied Bhagirathi valley zone is not subjected to pollution concerning PTEs and hence has a low potential for carcinogenic risk to the inhabitants in Garhwal Himalaya.

#### Non-carcinogenic and carcinogenic risks

##### Non-carcinogenic risk

To explore the effect of PTEs on human health, hazard risk assessment has also been carried out due to ingestion and dermal pathways on two sub-population groups, e.g. adults and children. The non-carcinogenic CDI and HQ values for ingestion and dermal routes about each analyzed PTE for adults and children are presented in Tables 9 and 10. Similarly, the estimated HI values for ingestion and dermal routes for adults and children are shown in Table 9 and Fig. 5a and b. The human body exposure through oral and dermal pathways was estimated in terms of CDI_ingestion_ and HQ_ingestion_ values using Eqs. (6) and (8). Tables 9 and 10 indicates that for both population sub-groups, such as adults and children, the average HQ_ingestion_ values exhibited the following sequence: Al < Pb < Cu < Cd < Se < Zn < Mn < As < Cr. For adults, the HQ_ingestion_ values for Zn showed wide range (5.63 × 10^−4^ to 1.64 × 10^−1^) with a mean value of 2.14 × 10^−2^ ± 3.37 × 10^−2^. Similarly, in the case of children, a wide range (8.18 × 10^−4^ to 2.38 × 10^−1^) is also characterized by Zn with a mean value of 3.11 × 10^−2^ ± 4.90 × 10^−2^ (Table 9).

**Table 9:** Non-carcinogenic risk to human health due to the ingestion of various PTEs through drinking water.

Element	Statistical parameter	Elemental concentration (µg L^-1^)	Adults		Children	
			Chronic daily intake (CDI)	Hazard quotient HQ)	chronic daily intake (CDI)	Hazard quotient (HQ)
Al	Min	0.39	1.83 × 10^−2^	1.83 × 10^−5^	2.67 × 10^−2^	2.67 × 10^−5^
	Max	17.45	8.25 × 10^−1^	8.25 × 10^−4^	1.20	1.20 × 10^−3^
	AM	3.09	1.46 × 10^−1^	1.46 × 10^−4^	2.12 × 10^−1^	2.12 × 10^−4^
	SD	3.79	1.79 × 10^−1^	1.79 × 10^−4^	2.60 × 10^−1^	2.60 × 10^−4^
Cr	Min	3.30	1.56 × 10^−1^	5.19 × 10^−2^	2.26 × 10^−1^	7.55 × 10^−2^
	Max	9.71	4.59 × 10^−1^	1.53 × 10^−1^	6.67 × 10^−1^	2.22 × 10^−1^
	AM	5.86	2.77 × 10^−1^	9.24 × 10^−2^	4.03 × 10^−1^	1.34 × 10^−1^
	SD	1.76	8.32 × 10^−2^	2.77 × 10^−2^	1.21 × 10^−1^	4.03 × 10^−2^
Mn	Min	0.08	3.97 × 10^−3^	1.65 × 10^−4^	5.77 × 10^−3^	2.40 × 10^−4^
	Max	178.44	8.43	3.51 × 10^−1^	1.23 × 10^+1^	5.11 × 10^−1^
	AM	13.31	6.29 × 10^−1^	2.62 × 10^−2^	9.14 × 10^−1^	3.81 × 10^−2^
	SD	37.45	1.77	7.37 × 10^−2^	2.57	1.07 × 10^−1^
Cu	Min	0.42	1.98 × 10^−2^	4.96 × 10^−4^	2.88 × 10^−2^	7.21 × 10^−4^
	Max	10.14	4.79 × 10^−1^	1.20 × 10^−2^	6.96 × 10^−1^	1.74 × 10^−2^
	AM	2.79	1.32 × 10^−1^	3.30 × 10^−3^	1.92 × 10^−1^	4.79 × 10^−3^
	SD	2.52	1.19 × 10^−1^	2.97 × 10^−3^	1.73 × 10^−1^	4.32 × 10^−3^
Zn	Min	3.57	1.69 × 10^−1^	5.63 × 10^−4^	2.45 × 10^−1^	8.18 × 10^−4^
	Max	1041.44	4.92 × 10^+1^	1.64 × 10^−1^	7.15 × 10^+1^	2.38 × 10^−1^
	AM	135.72	6.41	2.14 × 10^−2^	9.32	3.11 × 10^−2^
	SD	214.08	1.01 × 10^+1^	3.37 × 10^−2^	1.47 × 10^+1^	4.90 × 10^−2^
As	Min	0.31	1.48 × 10^−2^	4.95 × 10^−3^	2.16 × 10^−2^	7.19 × 10^−3^
	Max	19.73	9.33 × 10^−1^	3.11 × 10^−1^	1.36	4.52 × 10^−1^
	AM	3.20	1.51 × 10^−1^	5.03 × 10^−2^	2.19 × 10^−1^	7.32 × 10^−2^
	SD	3.73	1.76 × 10^−1^	5.87 × 10^−2^	2.56 × 10^−1^	8.54 × 10^−2^
Se	Min	0.20	9.59 × 10^−3^	1.92 × 10^−3^	1.39 × 10^−2^	2.79 × 10^−3^
	Max	1.97	9.29 × 10^−2^	1.86 × 10^−2^	1.35 × 10^−1^	2.70 × 10^−2^
	AM	0.64	3.01 × 10^−2^	6.01 × 10^−3^	4.37 × 10^−2^	8.74 × 10^−3^
	SD	0.36	1.72 × 10^−2^	3.44 × 10^−3^	2.50 × 10^−2^	5.01 × 10^−3^
Cd	Min	0.01	5.67 × 10^−4^	1.13 × 10^−3^	8.24 × 10^−4^	1.65 × 10^−3^
	Max	0.14	6.66 × 10^−3^	1.33 × 10^−2^	9.69 × 10^−3^	1.94 × 10^−2^
	AM	0.04	1.90 × 10^−3^	3.80 × 10^−3^	2.76 × 10^−3^	5.52 × 10^−3^
	SD	0.03	1.40 × 10^−3^	2.80 × 10^−3^	2.03 × 10^−3^	4.06 × 10^−3^
Pb	Min	0.01	3.78 × 10^−4^	1.08 × 10^−4^	5.50 × 10^−4^	1.57 × 10^−4^
	Max	0.37	1.73 × 10^−2^	4.94 × 10^−3^	2.51 × 10^−2^	7.18 × 10^−3^
	AM	0.04	1.95 × 10^−3^	5.57 × 10^−4^	2.83 × 10^−3^	8.09 × 10^−4^
	SD	0.06	2.90 × 10^−3^	8.27 × 10^−4^	4.21 × 10^−3^	1.20 × 10^−3^

**Table 10:** Non-carcinogenic risk to human health due to the dermal exposure of various PTEs through potable groundwater.

Element	Statistical parameter	Elemental concentration (µg L^-1^)	Adult		Children	
			Chronic daily intake (CDI)	Hazard quotient (HQ)	Chronic daily intake (CDI)	Hazard quotient (HQ)
Al	Min	0.39	5.55 × 10^−5^	5.55 × 10^−8^	8.80 × 10^−5^	8.80 × 10^−8^
	Max	17.45	2.50 × 10^−3^	2.50 × 10^−6^	3.96 × 10^−3^	3.96 × 10^−6^
	AM	3.09	4.42 × 10^−4^	4.42 × 10^−7^	7.00 × 10^−4^	7.00 × 10^−7^
	SD	3.79	5.42 × 10^−4^	5.42 × 10^−7^	8.59 × 10^−4^	8.59 × 10^−7^
Cr	Min	3.30	9.43 × 10^−5^	1.26 × 10^−3^	7.47 × 10^−4^	9.96 × 10^−3^
	Max	9.71	2.78 × 10^−4^	3.70 × 10^−3^	2.20 × 10^−3^	2.93 × 10^−2^
	AM	5.86	1.68 × 10^−4^	2.24 × 10^−3^	1.33 × 10^−3^	1.77 × 10^−2^
	SD	1.76	5.04 × 10^−5^	6.72 × 10^−4^	3.99 × 10^−4^	5.32 × 10^−3^
Mn	Min	0.08	1.20 × 10^−5^	1.25 × 10^−5^	1.90 × 10^−5^	1.98 × 10^−5^
	Max	178.44	2.55 × 10^−2^	2.66 × 10^−2^	4.04 × 10^−2^	4.21 × 10^−2^
	AM	13.31	1.90 × 10^−3^	1.98 × 10^−3^	3.02 × 10^−3^	3.14 × 10^−3^
	SD	37.45	5.36 × 10^−3^	5.58 × 10^−3^	8.49 × 10^−3^	8.84 × 10^−3^
Cu	Min	0.42	6.01 × 10^−5^	1.50 × 10^−6^	9.52 × 10^−5^	2.38 × 10^−6^
	Max	10.14	1.45 × 10^−3^	3.62 × 10^−5^	2.30 × 10^−3^	5.75 × 10^−5^
	AM	2.79	3.99 × 10^−4^	9.98 × 10^−6^	6.33 × 10^−4^	1.58 × 10^−5^
	SD	2.52	3.60 × 10^−4^	9.00 × 10^−6^	5.70 × 10^−4^	1.43 × 10^−5^
Zn	Min	3.57	5.11 × 10^−4^	1.70 × 10^−6^	8.09 × 10^−4^	2.70 × 10^−6^
	Max	1041.44	1.49 × 10^−1^	4.96 × 10^−4^	2.36 × 10^−1^	7.87 × 10^−4^
	AM	135.72	1.94 × 10^−2^	6.47 × 10^−5^	3.08 × 10^−2^	1.03 × 10^−4^
	SD	214.08	3.06 × 10^−2^	1.02 × 10^−4^	4.85 × 10^−2^	1.62 × 10^−4^
As	Min	0.31	4.49 × 10^−5^	1.50 × 10^−5^	7.12 × 10^−5^	2.37 × 10^−5^
	Max	19.73	2.82 × 10^−3^	9.41 × 10^−4^	4.47 × 10^−3^	1.49 × 10^−3^
	AM	3.20	4.57 × 10^−4^	1.52 × 10^−4^	7.24 × 10^−4^	2.41 × 10^−4^
	SD	3.73	5.33 × 10^−4^	1.78 × 10^−4^	8.45 × 10^−4^	2.82 × 10^−4^
Se	Min	0.20	2.90 × 10^−5^	5.81 × 10^−6^	4.60 × 10^−5^	9.20 × 10^−6^
	Max	1.97	2.81 × 10^−4^	5.62 × 10^−5^	4.46 × 10^−4^	8.91 × 10^−5^
	AM	0.64	9.10 × 10^−5^	1.82 × 10^−5^	1.44 × 10^−4^	2.88 × 10^−5^
	SD	0.36	5.21 × 10^−5^	1.04 × 10^−5^	8.26 × 10^−5^	1.65 × 10^−5^
Cd	Min	0.01	1.72 × 10^−6^	1.37 × 10^−4^	2.72 × 10^−6^	2.18 × 10^−4^
	Max	0.14	2.02 × 10^−5^	1.61 × 10^−3^	3.20 × 10^−5^	2.56 × 10^−3^
	AM	0.04	5.74 × 10^−6^	4.59 × 10^−4^	9.10 × 10^−6^	7.28 × 10^−4^
	SD	0.03	4.23 × 10^−6^	3.38 × 10^−4^	6.71 × 10^−6^	5.36 × 10^−4^
Pb	Min	0.01	1.14 × 10^−6^	3.27 × 10^−7^	1.81 × 10^−6^	5.18 × 10^−7^
	Max	0.37	5.23 × 10^−5^	1.50 × 10^−5^	8.30 × 10^−5^	2.37 × 10^−5^
	AM	0.04	5.90 × 10^−6^	1.69 × 10^−6^	9.35 × 10^−6^	2.67 × 10^−6^
	SD	0.06	8.76 × 10^−6^	2.50 × 10^−6^	1.39 × 10^−5^	3.97 × 10^−6^

However, none of the studied water samples is characterized by a high HQ_ingestion_ value greater than the unity in Table 9. Therefore, without any exception, the HQ_ingestion_ of studied groundwater sources corresponding to each analyzed PTE is estimated to be less than 1. Further, the HQ_ingestion_ values in children are always high compared to those of adults (Table 9).

Further, CDI_dermal_ and HQ_dermal_ values were also calculated using Eqs. (7) and (9), respectively and the values of HQ_der_ values corresponding to nine PTEs are presented in Table 10**.** It is observed that the HQ_der_ values corresponding to Mn exhibits wide variation **(**Table 10**).** Further, none of the analyzed PTEs is observed to be greater than unity. Thus, there are negligible potential human health risks from groundwater sources in the Bhagirathi valley of the Garhwal Himalaya region that is contaminated with dissolved PTEs.

**Figure 6:**
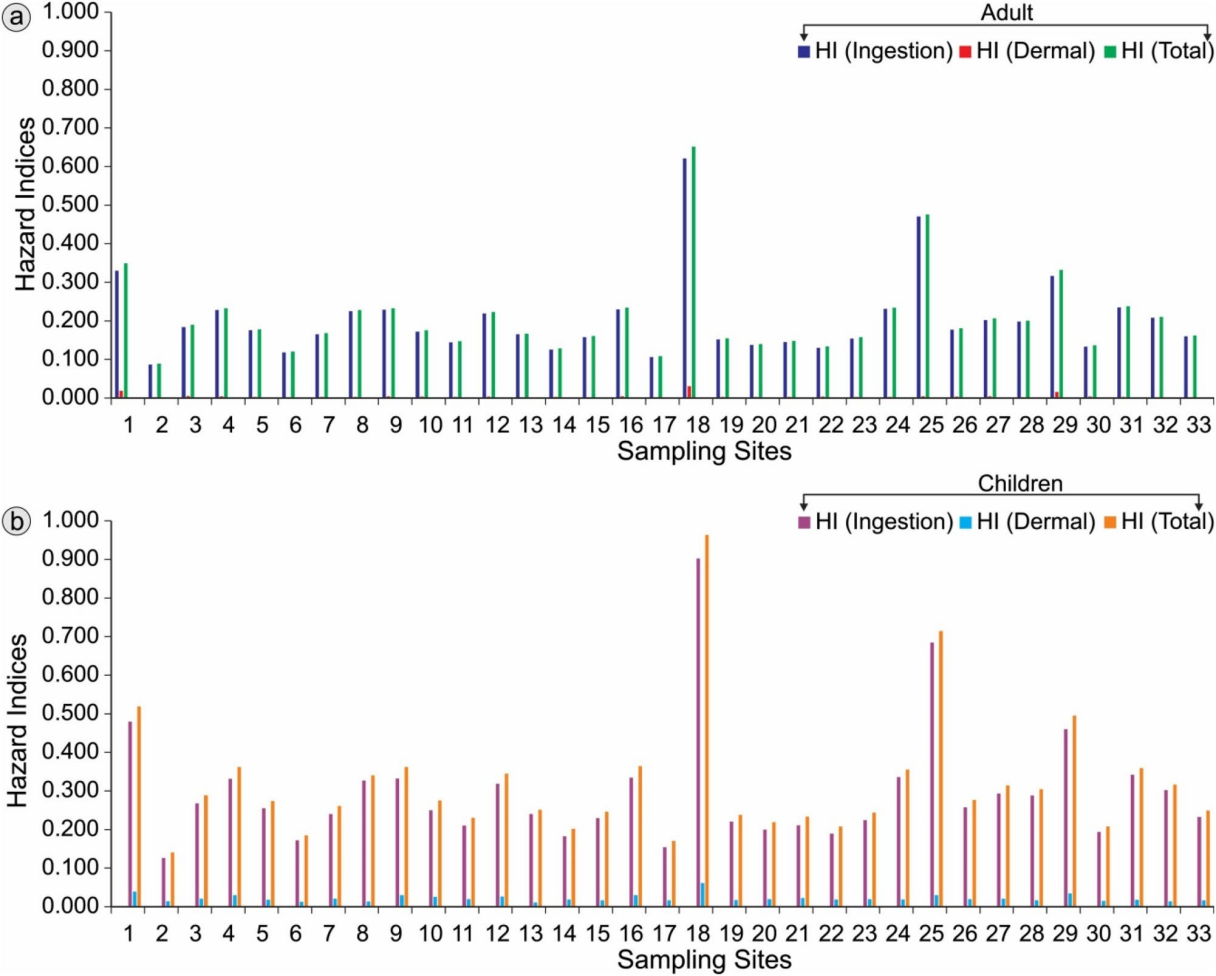
Variation of hazard indices of PTEs in potable groundwater of study area for **a** adults and **b** children.

Human exposure to multiple chemicals was estimated in terms of hazard index (HI). The HI is the measure of an overall effect posed by analyzed non-carcinogenic PTEs via ingestion and dermal pathways. Figure 6a and b exhibit the HI_ing_ and HI_der_ values for each sampling location corresponding to analyzed PTEs of the groundwater sources. The estimated HI_ing_ and HI_der_ were observed < 1, showing insignificant risks to the inhabitants of the study area. Moreover, based on obtained HI_ing_ and HI_der_ values, the total Hazard Index (HI_tot_) was further estimated by adding together all HIs via ingestion and dermal modes. The estimated average values of HI_ing_, HI_der_ and HI_total_ obtained for adults were found to be 0.204, 0.005, and 0.209, respectively. These indices were obtained as 0.297, 0.022 and 0.319 for children in the study zone. The high values of HI at sites 18 and 25 are due to high concentrations of Mn and As, respectively (Table 11).

**Table 11:** Statistical values of estimated HI_ing_, HI_der_, and HI_tot_ for adults and children due to PTEs contamination through water in the study area.

Statistical parameter	HI_ing_		HI_der_		HI_tot_	
	Adult	Children	Adult	Children	Adult	Children
Min	0.087	0.126	0.002	0.011	0.089	0.140
Max	0.621	0.903	0.031	0.061	0.652	0.964
AM	0.204	0.297	0.005	0.022	0.209	0.319
SD	0.103	0.150	0.006	0.009	0.108	0.158
GM	0.187	0.271	0.004	0.021	0.191	0.293

##### Carcinogenic risk

The estimated carcinogenic human health risks (CR_ing_ and CR_der_) associated with analyzed PTEs viz., Cr, As, Cd, and Pb in groundwater sources are shown in Table 12. The carcinogenic risks associated with other analyzed PTEs were not estimated due to the unavailability of corresponding cancer slope factors (CSFs).

**Table 12:** The estimated carcinogenic health risk from exposure to Cr, As, Cd and Pb in drinking water via ingestion and dermal routes.

Element	Statistical parameter	Ingestion route		Dermal route	
		Adults	Children	Adults	Children
Cr	Min	2.34 × 10^−1^	7.42 × 10^−3^	2.16 × 10^−4^	3.21 × 10^−6^
	Max	6.88 × 10^−1^	4.66 × 10^−1^	2.53 × 10^−3^	1.47 × 10^−4^
	AM	4.16 × 10^−1^	7.55 × 10^−2^	7.21 × 10^−4^	1.66 × 10^−5^
	SD	1.25 × 10^−1^	8.81 × 10^−2^	5.31 × 10^−4^	2.46 × 10^−5^
	GM	3.98 × 10^−1^	4.57 × 10^−2^	6.06 × 10^−4^	1.07 × 10^−5^
As	Min	3.40 × 10^−1^	1.08 × 10^−2^	3.13 × 10^−4^	4.67 × 10^−6^
	Max	1.00	6.78 × 10^−1^	3.68 × 10^−3^	2.14 × 10^−4^
	AM	6.04 × 10^−1^	1.10 × 10^−1^	1.05 × 10^−3^	2.41 × 10^−5^
	SD	1.81 × 10^−1^	1.28 × 10^−1^	7.72 × 10^−4^	3.58 × 10^−5^
	GM	5.79 × 10^−1^	6.64 × 10^−2^	8.81 × 10^−4^	1.56 × 10^−5^
Cd	Min	1.41 × 10^−4^	2.25 × 10^−5^	6.52 × 10^−7^	9.72 × 10^−9^
	Max	4.16 × 10^−4^	1.41 × 10^−3^	7.66 × 10^−6^	4.45 × 10^−7^
	AM	2.52 × 10^−4^	2.28 × 10^−4^	2.18 × 10^−6^	5.01 × 10^−8^
	SD	7.56 × 10^−5^	2.67 × 10^−4^	1.61 × 10^−6^	7.45 × 10^−8^
	GM	2.41 × 10^−4^	1.38 × 10^−4^	1.83 × 10^−6^	3.24 × 10^−8^
Pb	Min	1.12 × 10^−3^	1.57 × 10^−1^	1.03 × 10^−6^	1.54 × 10^−8^
	Max	3.30 × 10^−3^	9.87	1.21 × 10^−5^	7.05 × 10^−7^
	AM	1.99 × 10^−3^	1.60	3.46 × 10^−6^	7.95 × 10^−8^
	SD	5.99 × 10^−4^	1.86	2.55 × 10^−6^	1.18 × 10^−7^
	GM	1.91 × 10^−3^	9.67 × 10^−1^	2.91 × 10^−6^	5.14 × 10^−8^

The cancerous risks are considered safe in case of ≤ 1.0 × 10^−6^ according to the prescription limit of USEPA^47^ for various PTEs. The computed CR_ing_ values for adults follow the sequence: As > Cr > Pb > Cd, while dominance for children sub-group under the same ingestion category is found in the frequency: Cd < Cr < As < Pb. The adults may be at high cancer risk due to daily dermal exposure route to the high values of As, Cr, Pb, and Cd through groundwater. The potential risks ascribed due to As and Cr through the dermal pathway for children are high owing to value (> 1.0 × 10^−6^) due to their carcinogenic natures displayed in Table 12. The carcinogen to children only due to high levels of As and Cr. The estimated high CR_ing_ and CR_der_ values (> 1.0 × 10^−6^) may suggest an individual's lifetime risk of acquiring cancer^47,81^. For example, CR_ing_ and CR_der_ of 10^−4^ indicate that in cancer may be developed in 1 among 10,000 people^64^. Furthermore, children are more susceptible to Pb ingestion through drinking water.

### Removal of PTEs from drinking water

Numerous studies are reported on the primary investigation of the prevalence of PTEs in potable water sources of different underdeveloped nations^34^. Significant research has been carried out on treatment techniques of removing PTEs including chlorination, adsorption, ion-exchange, boiling, and solar disinfection. However, some of them are not much effective for PTEs removal^96^. Removing PTEs from potable water is essential to protect human health. Several studies exist on the removal of on As, Cd, Cr, Cu, Fe, Hg, Ni, Pb, and Zn^46,48^. The treatment techniques e.g. adsorption, chemical precipitation, electrochemical, ion exchange, membrane filtration, biological, hybrid method etc. are recognized as effective methods for PTEs removal from water^97,98^. Among these, adsorption is trusted to be advantageous for PTEs removal from water^99^. The combined approach of chemical precipitation and biological treatment performed by Ahmed et al.^98^ report a successful recovery of 99.3 and 98.4% of total Cr and Cr(VI), respectively from tannery effluent. The coagulation-flocculation for PTEs removal emphasized that apart from Cu^2+^, Pb^2+^, and Ni^2+^, other metals such as As^2+^, Se^2+^, Cr^2+^, Sb^3+^, Sb^5+^, Ag^2+^ also removed efficiently^100^.

It is noteworthy that some methods, e.g., ion exchange, membrane filtration, etc. are not feasible in low- and medium-income states owing to high cost. For such countries, the proposed treatment technologies should be less costly and also easy to adopt. Further, it should be developed using readily available local resources by local workers so that less skilled persons can also handle the system. Moreover, such treatment systems should require low operating and maintenance costs. All the above discussed approaches are having their own pro and cons depending upon local factors. However, among these, adsorption process has been considered relatively better than others in terms of cost and easy handling. Further, low-cost adsorbent development is still a challenge for the scientific society and it requires extensive research yet^101^. However, ion exchange can also be good for the removal of PTEs, if high costs can be beard. Otherwise adsorption can be given preference over most of the water purification techniques after considering local needs and challenges.

## Conclusions and recommendations

Based on the present work’s findings, it was found that the concentrations of PTEs viz. Al, Cr, Cu, Zn, Se, Cd, and Pb were not present above their acceptable limits prescribed by the BIS and WHO in the water of studied area for drinking purpose. However, the concentrations of Mn and As in a few samples were found to be greater than their prescribed limits. The relatively high values of Mn in groundwater may be ascribed to the Mn-containing agrochemicals release of untreated domestic sewage sludge in the study area. On the other hand, high value of As in groundwater may be attributed to pipeline corrosion and groundwater dynamics. The estimated values of pollution indices (HPI, HEI, and CI) were observed well below the threshold values which indicates groundwater is not polluted in the investigated region. However, if the partially treated or untreated domestic and municipal sewages are continuously discharged, need of a effective and efficient water treatment becomes mandatory to check the movement of toxic elements into the groundwater aquifer. The present study is an indicative work in nature as a pilot project and it is very significant in terms of public health protection. One of the limitations of this study is the absence of uncertainty analysis in the human health risk assessment, which could have provided valuable insights into the reliability and robustness of the risk estimates. Therefore, we recommend that future studies in this field incorporate uncertainty analysis to enhance the accuracy and comprehensiveness of risk assessments. In this geographical area, the floating population of pilgrimage is very high so that it is vital to conduct such type of studies in near future. . Thus, it can be inferred that such type of pilot research works can be the basis for the future extensive studies. The results presented in this study can provide better insight to the local administration, government, public water supply agencies to ensure the optimum water quality of the study area.

## Data Availability

The data generated and analyzed in this study are available from the corresponding author upon reasonable request.
